# Dietary Supplementation With Xylo-oligosaccharides Modifies the Intestinal Epithelial Morphology, Barrier Function and the Fecal Microbiota Composition and Activity in Weaned Piglets

**DOI:** 10.3389/fvets.2021.680208

**Published:** 2021-06-16

**Authors:** Jiayi Su, Wanghong Zhang, Cui Ma, Peifeng Xie, Francois Blachier, Xiangfeng Kong

**Affiliations:** ^1^Hunan Provincial Key Laboratory of Animal Nutritional Physiology and Metabolic Process, Chinese Academy of Sciences Key Laboratory of Agro-Ecological Processes in Subtropical Region, National Engineering Laboratory for Pollution Control and Waste Utilization in Livestock and Poultry Production, Institute of Subtropical Agriculture, Chinese Academy of Sciences, Changsha, China; ^2^Université Paris-Saclay, AgroParisTech, INRAE, UMR PNCA, Paris, France

**Keywords:** colon barrier function, intestine morphology, metabolites, microbiota, weaned piglets, xylo-oligosaccharides

## Abstract

The present study determined the effects of dietary xylo-oligosaccharides (XOS) supplementation on the morphology of jejunum and ileum epithelium, fecal microbiota composition, metabolic activity, and expression of genes related to colon barrier function. A total of 150 piglets were randomly assigned to one of five groups: a blank control group (receiving a basal diet), three XOS groups (receiving the basal diet supplemented with 100, 250, and 500 g/t XOS, respectively), as well as a positive control group, used as a matter of comparison, that received the basal diet supplemented with 0.04 kg/t virginiamycin, 0.2 kg/t colistin, and 3,000 mg/kg ZnO. The trial was carried out for 56 days. The results showed that the lowest dose tested (100 g/t XOS) increased (*P* < 0.05) the ileal villus height, the relative amount of *Lactobacillus* and *Bifidobacterium* spp., and the concentration of acetic acid and short-chain fatty acid in feces when compared with the blank control group. In conclusion, dietary 100 g/t XOS supplementation modifies the intestinal ecosystem in weaned piglets in an apparently overall beneficial way.

## Introduction

Weaning is a critical stage for piglets that is associated with alterations in the morphology, architecture, and function of the gut, as well as changes in the enteric microbiota (1). Weaned piglets consistently exhibit underdeveloped immune systems, digestive disorders, and post-weaning diarrhea (2), all of which decrease growth performance and cause economic loss for the swine industry (3). Antibiotics and ZnO have long been incorporated into the diets of piglets to solve post-weaning problems (4), maybe by ameliorating the intestinal disorders that are concomitant with weaning. However, their continuous use and misuse have led to the emergence of drug resistance, the risk of residual antibiotics in animal products, and Zn-residues in the environment (5, 6). In addition, the use of antibiotics in feed has been banned in China since 2020 to avoid development of antibiotic resistance among pathogenic microorganisms (7). Moreover, the country has also limited the amount of zinc oxide in feed. These regulations promoted the exploration of natural plant bioactive compounds, probiotics, prebiotics, and other potential alternatives as feed additives to improve productivity, welfare, and health of livestock and poultry (8).

Natural plant bioactive compounds can improve barrier function and nutrient transport in the gastrointestinal and have antimicrobial, antioxidant, and various pharmacological effects (8). The consumption of probiotic bacteria contributes to intestinal function by maintaining paracellular permeability, enhancing the physical mucous layer, stimulating the immune system, and modulating resident microbiota composition and activity (9). Prebiotics are compounds that, from an overall point of view, have the potential to influence positively some physiological functions, consequently, animal health, and well-being. Prebiotics may affect specifically and selectively the intestinal bacteria composition and/or metabolic activity (10). Dietary modulation of the gut microflora by prebiotics is designed to improve health, and notably gut health, by stimulating numbers and/or activities of the so-called beneficial bacteria, such as the *Bifidobacterium* and *Lactobacilli*. These beneficial bacteria may contribute to the increased resistance to pathogenic bacteria and the stimulation of the immune response (11).

It is well-known that, for instance, β-(2-1)-fructans, which include inulin and fructo-oligosaccharides (FOS), are considered as truly prebiotics, while xylo-oligosaccharides (XOS) are considered as candidate prebiotics (12). The XOS is a functional polymerized sugar made up of 2-8 xylose molecules bonded by β-(1–4) glycosidic bounds. In addition to xylose molecules, XOS is usually found in combination with other side groups such as α-D-glucopyranosyl uronic acid or its 4-O-methyl derivative, acetyl groups, or arabinofuranosyl residues, resulting in branched XOS with diverse biological properties (10).

As pigs do not have endogenous enzymes in the small intestine to digest XOS, it is expected that unabsorbed XOS can reach the hindgut where they will be used for the growth of favorable bacteria and be fermented by the microbiota (13) leading to the production of various metabolites. Some studies have shown that XOS preferentially stimulates the growth or activity of advantageous bacteria such as *Bifidobacterium* and other lactic acid bacteria in the gastrointestinal tract (14–16). Liu et al. (17) found that XOS supplementation may increase slightly ADFI, as well as the digestive enzyme trypsin and amylase activity, and fecal microbial shedding of *Lactobacilli*, while decreasing fecal *E. coli* counts. Chen et al. (18) have reported that dietary XOS supplementation enhanced the growth performance, improved intestinal morphology and modulated the relative abundance of specific bacteria by changing the overall microbial composition and bacterial metabolite production. The increased population of *Lactobacillus* and decreased abundance of *Clostridium_sensu_stricto_1, Escherichia_Shigella*, and *Terrisporobacter* in piglets fed XOS 500 might be a contributor for improved growth characteristics (18).

Our previous study showed that XOS can significantly improve the growth of weaned pigs (19). The inclusion of XOS in weaned pigs' diets may enhance immune function and improve the growth of the intestinal mucosa layer and intestinal microbiota diversity (19). We hypothesized that dietary supplementation with XOS may exert beneficial effects on the jejunum and ileum morphology, fecal microbiota composition and bacterial metabolite concentrations, as well as expression of genes related to intestinal barrier function and cytokine production in colon of weaned piglets. Therefore, the aim of this study was to test this hypothesis and to determine what dose was most beneficial for the intestinal health of the weaned piglets.

## Materials and Methods

### Animals, Housing, and Treatment

A total of 150 Duroc × Landrace × Large White piglets weaned at 21-day-old with an average body weight of 7.02 ± 0.05 kg were used in this study. The piglets were randomly assigned to one of five groups with six replicates per group and five piglets per replicate. The five groups consisted of a blank control group (receiving a basal diet), three groups receiving the basal diet supplemented with 100, 250, or 500 g/t XOS group (XOS is mixed in the basal diet as one of feed additives), as well as a comparative group receiving a classical mixture of compounds (the basal diet supplemented with 0.04 kg/t virginiamycin, 0.2 kg/t colistin, and 3,000 mg/kg ZnO). The XOS were provided by Shandong Longlive Bio-technology Co. Ltd (Shandong, China), which contain xylobiose, xylotriose, and xylotetraose at ≥35%. The doses used for supplementation was according to the manufacturer's recommendations.

All pigs were housed in 2.0 m × 2.5 m pens with hard plastic slatted flooring and had *ad libitum* access to drinking water and the experimental diets. Each pen was equipped with a stainless-steel feeder and a nipple drinker. The room temperature was maintained at 25–27°C. The composition and nutrient levels of the basal diet are shown in Table 1. The trial lasted for 56 days.

**Table 1:** Composition and nutrient levels of the basal diet (fed-basis).

**Ingredients**	**Stages of the trial**		
	**7–28 days**	**29–40 days**	**41–56 days**
Corn	55.0	220.0	695.0
Extruded corn	100.0	–	–
Broken rice	180.0	250.0	–
Wheat flour	50.0	120.0	–
Glucose	50.0	30.0	–
Soybean meal (46% CP)	30.0	105.0	–
Soybean meal (43% CP)	–	–	160.0
Puffed soybean	100.0	100.0	–
Fermented soybean meal	–	25.0	40.0
Soybean protein concentrate	25.0	–	20.0
Fish meal	50.0	30.0	10.0
Plasma	50.0	–	–
Low-protein whey powder	225.0	50.0	–
Egg powder	10.0	5.0	–
Wheat bran	–	–	20.0
Soybean oil	20.0	10.0	15.0
Citric acid	15.0	15.0	–
Premix^a^	40.00	40.00	40.00
Total	1000.0	1000.0	1000.0
Nutrient component^b^			
DE (MJ/kg)	14.75	14.23	13.81
CP	18.43	18.02	16.59
EE	5.71	4.37	4.47
CF	1.68	2.31	2.76
Met	0.52	0.49	0.39
Cys	0.27	0.28	0.31

a *The premix provided the following per kg of the diet: VA 6 200 IU, VD3 700 IU, VE 88 IU, VK 4.4 mg, VB2 8.8 mg, Pantothenate 24.2 mg, nicotinic acid 33 mg, Chloride choline 330 mg, Cu 10 mg, Zn 100 mg, Fe 145 mg, Mn 40 mg, Se 0.1 mg, I 0.3 mg*.

b *Nutrient contents are calculated values. DE, digestible energy; CP, crude protein; EE, ether extract; CF, crude fiber; Lys, lysine; Met, methionine; Cys, cysteine*.

### Sample Collection and Preparation

At days 7, 21, and 56 of the trial, the feces were collected and stored at −20°C for analysis of short-chain fatty acids (SCFA) and of the microbiota composition. At the end of the 56-day trial period and 12 h after the last feeding, a medium-sized piglet per replicate was sacrificed using general anesthesia with Zoletil (15 mg tiletamine/kg body weight, 15 mg zolazepam/kg body weight, intramuscular injection) (20). After intestine recovery, samples of the jejunum, ileal, and colonic samples (~2 cm) were collected after washing with cold physiological saline, the jejunum and ileum were immediately fixed in 10% neutral formalin solution until further morphological analysis, and colon samples were frozen in liquid nitrogen and then stored at −80°C until gene expression analysis.

### The Measurement of Jejunal and Ileal Morphology

Histological slides were prepared from 3 cross-sections (5 μm thick) of each intestinal sample, which were processed in low-melt paraffin and stained with hematoxylin-eosin. Villus height (VH) and crypt depth (CD) were measured using the Olympus BX-51 microscope (Olympus, Center Valley, PA, USA), and VH:CD (V/C) ratio were calculated.

### DNA Extraction and Fecal Microbiota Analysis

Total microbial DNA was extracted and purified using a QIAamp DNA Stool Kit (Qiagen, Hilden, Germany) and stored at −80°C. The 16S rRNA gene sequences of *Bacteroidetes, Bifidobacterium* spp., *Escherichia coli*, and *Lactobacillus* were cloned into the pMD19-T vector (21). Gene sequences were amplified from fecal total DNA using the primers listed in Table 2. A total of five clones with 16S rRNA gene sequences belonging to different taxa were used as templates to test primer specificity. Standard curves were constructed with DNA from representative species for a concentration range of 10^2^–10^10^ DNA copies/mL using a Lightcycler 480II instrument (Applied Biosystems, Carlsbad, CA, USA). The general microbial DNA extracted from feces and specific DNA from recombinant microbiota were quantified by RT-PCR. Reaction conditions were 2 min at 50°C, an initial denaturation step at 95°C for 5 min, and then 40 cycles of denaturation at 94°C for 20 s, primer annealing at a species-specific temperature for 30 s, and primer extension at 60°C for 1 min (22).

**Table 2:** 16S rRNA gene-targeted group-specific primers used in this study.

**Items**	**Sequence (5^**′**^–3^**′**^)**	**Amplicon length (bp)**
*Bacteroidetes*	F:GGARCATGTGGTTTAATTCGATGAT	126
	R:AGCTGACGACAACCATGCAG	
*Bifidobacterium* spp.	F:TCGCGTCYGGTGTGAAAG	128
	R:GGTGTTCTTCCCGATATCTACA	
*Escherichia coli*	F:CATGCCGCGTGTATGAAGAA	95
	R:CGGGTAACGTCAATGAGCAAA	
*Lactobacillus*	F:AGCAGTAGGGAATCTTCCA	345
	R:ATTCCACCGCTACACATG	
	R:ACGTCRTCCMCNCCTTCCTC	

### Analysis of the Fecal Concentrations of Metabolites

At time of euthanasia, the feces of each pigs were sampled, homogenized, and centrifuged at 1,000 × g for 10 min (23). A mixture of supernatant fluid and 25% metaphosphoric acid solution (1:0.25 ml) was prepared for determining the SCFA by gas chromatography (24).

### Analysis of mRNA Levels of Genes Related to Barrier Function in Colonic Tissues

The analysis of mRNA levels of genes related to barrier function in colonic tissues was conducted according to a previous study. Total RNA was isolated from colonic samples using the TRIzol reagent (Invitrogen, Carlsbad, CA, USA) and treated with DNAase. The RNA quality was checked by 1% agarose gel electrophoresis followed by staining with 10 μg/mL ethidium bromide. The OD260:OD280 ratio of extracted RNA was between 1.8 and 2.0. Reverse transcription was performed using a Prime Script RT reagent Kit with gDNA eraser (Takara, Dalian, China). The mRNA levels of the selected genes in the colon tissues were determined by real-time quantitative (RT-qPCR) as described previously (25). Selected genes were intestinal tight junction proteins, including *occludin, Claudin 2* and *Claudin 3*, and *zonula occludens-1* (*ZO-1*); *interleukin-10 (IL-10), IL-1*β, and *IL-8*. The primers used to amplify these genes are shown in Table 3. The relative expression was reported as a ratio of the expression of the target genes to that of beta-actin (β-actin), and data were expressed relative to those in the basal diet-treated piglets. The relative expression ratio (R) of mRNA was calculated as R = 2^−Δ*ΔCt*^ × (sample − control), where -ΔΔCt × (sample − control) = (Ct_target gene_ − Ct_β−*actin*_) × sample − (Ct_target gene_ − Ct_β−*actin*_) × control. RT-qPCR was performed using a SYBR Green detection kit (Thermo Fisher Scientific, Waltham, MA), and conditions were as follows: 30 s denaturation at 94°C, 30 s annealing at 60°C, and 30 s extension at 72°C for 40 cycles, followed by a melting curve program (60–99°C with a heating rate of 0.1°C/s and fluorescence measurement). A melting temperature (T_m_) peak at 85 ± 0.8°C was used to determine the specificity of amplification. T_m_ values are reported as the mean of three replicates.

**Table 3:** Primers used for quantitative reverse transcription PCR.

**Items**	**Genbank accession number**	**Sequence (5^′^–3^′^)**	**Length (bp)**
*β-actin*	XM_003357928.1	F:GGACTTCGAGCAGGAGATGG R:GCACCGTGTTGGCGTAGAGG	233
*Occludin*	NM_001163647.1	F:ATGCTTTCTCAGCCAGCGTA R:AAGGTTCCATAGCCTCGGTC	176
*Claudin 2*	NM_001161638	F:TGGTGGGTGGAGTCTTCTTC R: TTGGAGCGATTTCCTTGC	223
*Claudin 3*	NM_001160075	F:GATGCAGTGCAAAGTGTACGA R:GTCCTGCACGCAGTTGGT	148
*ZO-1*	XM_003353439.1	F:GAGGATGGTCACACCGTGGT R:GGAGGATGCTGTTGTCTCGG	169
*IL-1β*	NM_214055.1	F:AGTGGAGAAGCCGATGAAGA R:CATTGCACGTTTCAAGGATG	113
*IL-8*	NM_213867.1	F:TAGGACCAGAGCCAGGAAGA R:AGCAGGAAAACTGCCAAGAA	92
*IL-10*	NM_214041.1	F:CTGCCTCCCACTTTCTCTTG R:TCAAAGGGGCTCCCTAGTTT	95

*β, beta; IL, interleukin; ZO-1, zonula occludens-1*.

### Statistical Analysis

Results were statistically analyzed using one-way ANOVA of SPSS 17.0 software (SPSS, Inc., Chicago, IL, USA). The pen was included as a random effect for data analysis. The data were presented as means, pooled SEM, and *P*-values. *P*-values < 0.05 were taken to indicate statistical significance.

## Results

### The Morphology of the Epithelium of Jejunum and Ileum

As shown in Table 4, the CD and VH of jejunum in the 100 g/t XOS group increased (*P* < 0.05) in comparison to the positive control group. The V/C ratio of jejunum in the 500 g/t XOS group was higher (*P* < 0.05) than the blank control group. The VH of ileum in the 100 g/t XOS group increased (*P* < 0.05) in comparison to the other four groups.

**Table 4:** Effects of dietary XOS supplementation on the morphology of jejunal and ileal epithelium in weaned piglets (n = 6; data are mean values in μm).

**Items**	**Blank control group**	**XOS-supplemented groups (g/t)**			**Positive control group**	**Pooled SEM**	***P-*value**
		**100**	**250**	**500**			
**Jejunum**
Villus height	510.55^ab^	607.62^a^	484.20^b^	497.45^ab^	482.98^b^	47.28	0.03
Crypt depth	324.42^ab^	377.73^a^	297.89^b^	277.68^b^	297.08^b^	22.75	0.02
V:C ratio	1.58^b^	1.61^ab^	1.65^ab^	1.81^a^	1.63^ab^	0.07	0.04
**Ileum**
Villus height	418.70^b^	492.4^a^	389.53^b^	428.41^b^	401.92^b^	19.37	0.02
Crypt depth	219.66	259.76	222.78	242.92	221.87	18.42	0.44
V:C ratio	1.92	1.93	1.76	1.84	1.82	0.12	0.85

*Data in the same row with different superscripts differ significantly (P < 0.05). V:C ratio, Villus height:Crypt depth ratio*.

### Composition of Fecal Microbiota in Piglets

As shown in Table 5, the relative abundances of *Lactobacillus, Bifidobacterium* spp*., Escherichia coli*, and *Bacteroidetes* in the feces did not change (*P* > 0.05) after XOS supplementation at day 7 of the trial. In the 250 and 500 g/t XOS groups and the positive control group, the relative abundance of *Lactobacillus* was lower (*P* < 0.05) than the blank control group at day 21 of the trial. At day 56 of the trial, the relative abundance of *Lactobacillus* in the 100 g/t XOS group was higher (*P* < 0.05) than the blank control group. In the 100, 250, and 500 g/t XOS groups, the relative abundances of *Bifidobacterium* spp. was higher (*P* < 0.05) than the blank control group. In the 100 g/t XOS group, the relative abundance of *Bacteroidetes* was higher when compared to the positive control group.

**Table 5:** Effects of dietary XOS supplementation on fecal microbiota abundance in weaned piglets (*n* = 6; lg copies/g).

**Items**	**Blank control group**	**XOS-supplemented groups (g/t)**			**Positive control group**	**Pooled SEM**	***P-*value**
		**100**	**250**	**500**			
**Day 7**							
*Lactobacillus*	6.82	6.89	7.22	7.20	7.36	0.31	0.33
*Bifidobacterium* spp.	5.44	5.68	5.51	5.58	5.84	0.17	0.17
*Escherichia coli*	6.38	6.66	6.35	6.37	6.71	0.22	0.37
*Bacteroidetes*	10.60	10.79	10.68	10.69	10.76	0.26	0.47
**Day 21**							
*Lactobacillus*	7.90^a^	7.33^ab^	6.94^b^	6.57^b^	6.50^b^	0.28	0.02
*Bifidobacterium* spp.	5.78	5.84	5.91	5.22	5.54	0.11	0.27
*Escherichia coli*	6.14	5.84	6.12	6.16	6.23	0.11	0.64
*Bacteroidetes*	10.80	10.55	10.54	10.41	10.66	0.11	0.51
**Day 56**							
*Lactobacillus*	7.36^b^	7.84^a^	7.47^ab^	7.43^ab^	7.57^ab^	0.13	0.02
*Bifidobacterium* spp.	6.20^b^	7.15^a^	7.13^a^	7.14^a^	6.94^ab^	0.25	0.04
*Escherichia coli*	6.61	6.49	6.96	6.39	6.58	0.36	0.86
*Bacteroidetes*	10.90^ab^	11.10^a^	10.85^ab^	10.98^ab^	10.66^b^	0.11	0.01

*Data in the same row with different superscripts differ significantly (P < 0.05)*.

### Concentrations of Bacterial Metabolites in the Feces of Piglets

As shown in Table 6, at day 7 of the trial, the acetic acid concentration in 100, 250, and 500 g/t XOS groups and the positive control group were higher (*P* < 0.05) than the blank control group. Dietary supplementation with 250 g/t XOS increased (*P* < 0.05) the SCFA concentration compared with the blank control group. At day 21 of the trial, the concentration of isovaleric acid in the 100 g/t XOS group increased (*P* < 0.05) compared with the other four groups. At day 56 of the trial, the concentration of acetic acid and SCFA in the 100 g/t XOS group was higher (*P* < 0.05), as well as the butyric acid and SCFA in the 250 g/t XOS group, when compared with the two control groups.

**Table 6:** Effects of dietary XOS supplementation on concentrations of short-chain fatty acids in colonic contents of weaned piglets (*n* = 6; data are mean value in mg/g).

**Items**	**Blank control group**	**XOS-supplemented groups (g/t)**			**Positive control group**	**Pooled SEM**	***P-*value**
		**100**	**250**	**500**			
**Day 7**							
Acetic acid	2.26^b^	3.46^a^	3.85^a^	3.65^a^	3.96^a^	0.30	0.01
Propionic acid	1.79	1.76	1.93	1.54	1.69	0.25	0.89
Butyric acid	1.08	0.90	1.29	0.85	1.16	0.19	0.57
Valeric acid	0.19	0.24	0.17	0.09	0.17	0.04	0.38
Isobutyric acid	0.17	0.22	0.19	0.17	0.17	0.02	0.46
Isovaleric acid	0.25	0.29	0.29	0.25	0.25	0.05	0.95
Short-chain fatty acid	5.74^b^	6.87^ab^	7.72^a^	6.55^ab^	7.39^ab^	0.57	0.03
**Day 21**							
Acetic acid	3.33	3.43	3.27	3.33	3.66	0.31	0.93
Propionic acid	1.31	1.55	1.31	1.32	1.45	0.16	0.82
Butyric acid	1.13	1.51	1.32	1.10	1.15	0.20	0.65
Valeric acid	0.36	0.42	0.43	0.31	0.34	0.04	0.42
Isobutyric acid	0.36	0.38	0.36	0.29	0.34	0.03	0.61
Isovaleric acid	0.24^b^	0.40^a^	0.27^b^	0.24^b^	0.29^b^	0.03	0.04
Short-chain fatty acid	6.74	7.69	6.97	6.58	7.23	0.51	0.70
**Day 56**							
Acetic acid	2.30^b^	2.94^a^	2.82^ab^	2.66^ab^	2.29^b^	0.21	0.04
Propionic acid	1.16	1.42	1.55	1.29	1.25	0.14	0.29
Butyric acid	0.61^b^	0.90^ab^	1.16^a^	0.92^ab^	0.72^b^	0.10	0.04
Valeric acid	0.14	0.22	0.23	0.20	0.17	0.03	0.24
Isobutyric acid	0.08	0.09	0.11	0.12	0.09	0.02	0.63
Isovaleric acid	0.07	0.08	0.10	0.10	0.08	0.02	0.77
Short-chain fatty acid	4.37^b^	5.66^a^	5.97^a^	5.28^ab^	4.59^b^	0.43	0.04

*Data in the same row with different superscripts differ significantly (P < 0.05). Short-chain fatty acid, including acetic acid, ropionic acid, butyric acid, valeric acid, isobutyric acid, and isovaleric acid*.

### Expression of Genes Related to Intestinal Barrier Function and Cytokine in the Colon

As shown in Table 7, dietary supplementation with 100 g/t XOS increased (*P* < 0.05) the mRNA level of *Claudin 2* compared with the positive control group. Dietary supplementation with 250 g/t XOS increased (*P* < 0.05) the mRNA level of *ZO-1* compared with the blank control group.

**Table 7:** Effects of dietary XOS supplementation on colon mRNA levels related to epithelial cell barrier function and inflammation in weaned piglets (n = 6).

**Items**	**Blank control group**	**XOS-supplemented groups (g/t)**			**Positive control group**	**Pooled SEM**	***P-*value**
		**100**	**250**	**500**			
*Occludin*	1.00	0.87	1.32	0.77	1.51	0.25	0.31
*Claudin 2*	1.00^ab^	0.87^b^	1.55^ab^	1.28^ab^	1.79^a^	0.24	0.02
*Claudin 3*	1.00	0.81	1.27	0.72	0.83	0.26	0.62
*ZO-1*	1.00^b^	1.37^ab^	1.90^a^	1.47^ab^	1.61^ab^	0.22	0.03
*IL-1β*	1.00	1.16	1.65	1.35	1.10	0.20	0.11
*IL-8*	1.00	1.04	1.29	1.24	0.90	0.23	0.16
*IL-10*	1.00	1.22	1.26	1.16	1.10	0.34	0.67

*Data in the same row with different superscripts differ significantly (P < 0.05). IL, interleukin; ZO-1, zonula occludens-1; β, beta*.

## Discussion

Recently, the intestinal microbiota composition and related metabolic activities have emerged as important parameters affecting either positively or negatively “intestinal health” (26). Components such as microbiota composition, diversity and bacterial metabolite concentrations can affect the epithelial integrity, barrier function, immunity, and enteroendocrine peptides secretion. It is known that the intestinal microbiota has genomic characteristics that allow it to use undigested or partially digested nutrients, notably proteins and undigested polysaccharides, present in feedstuff. In return, it benefits the host metabolism by providing energy through the production of metabolites that can be utilized and absorbed by colonic cells (27). Conversely, some amino acid-derived bacterial metabolites have been shown to exert deleterious effects on colonic epithelial cells when present in excess (26). It has been demonstrated that regarding oligo-saccharides, numerous types of these compounds may represent a potential alternative to antibiotic treatment for contributing to the maintenance of microbial composition and/or metabolic activity with positive effect on gut health (28). Indeed, David et al. (29) reviewed the beneficial effects of different oligo-saccharides, including *N*-acetylglucosamine, oligo-fructose, lactulose, and various glycoproteins, as prebiotics to improve the gut health. Li et al. (30) found that XOS promotes the growth of *Bifidobacterium* and *Lactobacillus* in the gut and increases the SCFA content in these intestinal microbes, these effects in turn being associated with an improvement of the intestinal barrier function (30). In the present study, three doses of XOS were used according to the manufacturer's recommendations.

Gut villus structure and barrier integrity play an important role in intestinal function, including nutrient digestibility, absorption, and protecting against pathogen infection (31). An increase of villus height suggests a larger surface area capable of greater absorption of available nutrients (32). The villus height/crypt height ratio is used as a criterion to estimate the nutrient final digestion step and absorption capacity of the small intestine (33). Prebiotics were reported to improve growth *via* promoting nutrient absorption by improving intestinal structure (34). In this study, we found that dietary supplementation with 500 g/t XOS increased the villus height/crypt height ratio in jejunum. In addition, we found that dietary supplementation with 100 g/t XOS increased the villus height in ileum. This confirms the results obtained by Mourao et al. (35) showing a positive relationship between dietary oligosaccharides supplementation and higher intestinal villi in piglets. Chen et al. found that villus height and villus height:crypt depth ratio of the ileum in the 500 g/t XOS treatment group was significantly increased compared to the CON group. De Maesschalck et al. (36) found that supplementation of 0.5% XOS to the broiler feed significantly improved the villus height of the ileum. These reports are all converging in showing that XOS can improve intestinal structure, in accordance with our finding, however with different doses used. Additional works are required in order to better clarify the relationship between dose and parameters related to intestinal functions.

Gut microbiota are the resident microorganisms in the digestive tracts of animals and humans, which affect nutrient digestion and the bioconversion of food compounds in the host organisms (37). Oligosaccharides can be fermented in the large intestine by indigenous bacteria and are preferably used by probiotic bacteria (38). Prebiotics are non-digestible feed ingredients that are believed to escape digestion in the upper gut and that may selectively stimulate the growth of *Lactobacilli* or *Bifidobacterium* in colon, thereby possibly improving health (39). Our results indicated that 100 g/t XOS increased the growth of *Lactobacillus* and *Bacteroidetes*. What's more, 100, 250, and 500 g/t XOS increased the growth of *Bifidobacterium* spp. at day 56 of the trial. It confirms that XOS can improve gut microbiota communities. Increased level of *Lactobacillus* and *Bifidobacterium* spp. has been proposed to reinforce the epithelial barrier function against common pathogens. The mechanisms are likely to include the excretion of organic acids (e.g., lactic acid and acetic acid), competition for nutrients with deleterious bacteria, competition at gut receptor sites, and immune-modulation and formation of specific antimicrobial agents (40). Moreover, we found that the advantage of 100 g/t XOS in changing intestinal microecology was revealed by the analysis of the different kinetics time points, such analysis showing that the effects of XOS is a long-term process.

It is well-known that the SCFAs are main products of the microbial fermentation of complex carbohydrates (41). These SCFAs are the preferred energy source for the colonic epithelium and may stimulate colonocyte proliferation and influence various aspects of gut physiology. Most of functional oligosaccharides are not digested for lack of relevant enzymes, and reach the colon in their initial form, before being fermented by anaerobic bacteria (42). Such metabolic process produces SCFAs. The amounts and types of SCFA produced depend on the type of non-digestible oligosaccharides as well as on the composition of the intestinal microbial flora (43). Our results showed that 100 g/t XOS increase the concentrations of acetic acid and SCFAs in the colon at day 56 of the trial. We have known that the production of SCFA results in decrease of pH in the colon, such a decrease inhibiting the growth of certain pathogenic bacterium while stimulating the growth of the beneficial bacteria (44, 45). This also explains why the abundance of *Lactobacillus* and *Bifidobacterium* spp. increased at 56 days of the trial. The SCFAs have been noted to have immunomodulatory effects on colonic inflammation and suppress inflammatory cytokine secretion in cultured epithelial cells (46). Increased availability of butyric acid for the colonocyte may be associated with decreases of the inflammatory parameters (47). In the present study, 250 g/t XOS supplementation increased the concentration of butyric acid at day 56 of the trial, but did not regulate the mRNA levels of inflammatory cytokines (*IL-1*β, *IL-8*, and *IL-10*), which is not consistent with the findings presented above. The reason and significance needs further investigations.

The surface of the gut epithelium is protected by a layer of mucus, which is in constant contact with an abundant population of microbiota and their metabolites (48). One aspect of the intestinal barrier is related to an intact intestinal epithelium with functional junctional complex, consisting of tight junctions (TJ, including *occludin, claudin*, and *ZO-1*), adherens junctions (AJs, including *E-Cadherin* and *catenins*), gap junctions, and desmosomes (49). Such an intact epithelium is able to exclude the vast majority of the microbiota from access to the subepithelial cells in the lamina propria (49). Our results indicated that the 250 g/t XOS up-regulated the mRNA level of *ZO-1*. *ZO-1* serves as a scaffold protein and anchors tight junction strand proteins (50). Yin et al. (51) also have reported that dietary XOS markedly enhanced the mRNA level of *ZO-1* in the ileum. However, further work is needed to determine if such an increased expression of *ZO-1* is paralleled by a modification of the epithelial barrier function. Christensen et al. (52) have reported that the relative abundances of beneficial microbiota have been found a highly association with gut barrier. But the data of our study did not prove such an association. Moreover, no differences in the mRNA levels of *Occludin, Claudin 2*, and *Claudin 3* were recorded when comparisons between XOS-supplemented groups and blank control group were done, thus indicating that the effect of XOS on *ZO-1* gene expression among other genes coding for tight junction protein appears rather specific. Additional studies are obviously required to define the complex relationships between the different parameters modified by dietary XOS supplementation.

## Conclusions

The lowest dose of xylo-oligosaccharides tested (100 g/t) increases the villus height in ileum, the abundance of *Lactobacillus* and *Bifidobacterium* spp. and the concentrations of acetic acid and short-chain fatty acid. Thus, 100 g/t xylo-oligosaccharides supplementation impacts the ileal morphology and colon ecosystem in weaned piglets. According to our findings, we recommend supplementation with 100 g/t xylo-oligosaccharides to feed in order to obtain the most presumed beneficial effects on the gut ecosystem.

## Data Availability Statement

The datasets presented in this study can be found in online repositories. The names of the repository/repositories and accession number(s) can be found at: https://www.ncbi.nlm.nih.gov/genbank/.

## Ethics Statement

The animal study was reviewed and approved by Animal Care and Use Committee of Institute of Subtropical Agriculture, Chinese Academy of Sciences.

## Author Contributions

JS, WZ, CM, PX, and XK performed the experiments. JS, FB, and XK wrote the manuscript. JS and XK performed the statistical analysis. JS, WZ, and PX fed the animals. All authors reviewed the manuscript.

## Conflict of Interest

The authors declare that the research was conducted in the absence of any commercial or financial relationships that could be construed as a potential conflict of interest.

## Abbreviations

AJ: adherens junctions
IL: interleukin
RT-qPCR: real-time quantitative PCR
SCFA: short-chain fatty acids
TJ: tight junctions
XOS: xylo-oligosaccharides
ZO: zonula occludens.
